# Bone fracture detection—Can artificial intelligence replace doctors in orthopedic radiography analysis?

**DOI:** 10.3389/frai.2023.1223909

**Published:** 2023-08-01

**Authors:** Aariz Hussain, Areeba Fareed, Shafaq Taseen

**Affiliations:** Karachi Medical and Dental College and Abbasi Shaheed Hospital, Karachi, Sindh, Pakistan

**Keywords:** Artificial Intelligence (AI), deep machine learning, bone fracture, orthopedics, radiology, radiology and AI, bone fracture detecting AI

## 1. Introduction

Bone diseases, such as femoral neck injuries, knee osteoarthritis, and fractures, are common, with an estimated 1.71 billion patients suffering from musculoskeletal problems worldwide (Meena and Roy, 2022). The rate is expected to double in 30 years (Meena and Roy, 2022). Patients with fractures are a common emergency presentation and may be misdiagnosed at radiologic imaging (Meena and Roy, 2022). Furthermore, fracture classification is vital for determining the surgical method and restoring the patient's mobility, which takes a lot of time, exposing clinicians to mental stress in the emergency room (Cha et al., 2022). Therefore, proper and timely diagnosis and treatment of a fractured patient are crucial (Meena and Roy, 2022).

Nowadays, Artificial Intelligence (AI) is a growing field that has experienced exponential growth, especially in the past 5 years. Therefore, the interest of researchers, clinicians, and radiologists in AI continues to grow (Chen et al., 2022). Deep learning (DL), a specific subset of AI, has shown promising results in computed radiographs for fracture detection, classification of Osteoarthritis, bone age, as well as automated measurements of the lower extremities (Chen et al., 2022). AI is playing an ever-increasing role in radiology to the extent that there are unfounded fears it will completely take over the radiologist's role (Offiah, 2022).

## 2. How does AI work?

AI uses advanced algorithms and computational models to process and analyze data. The process of developing and deploying AI systems involves several key stages. It starts with data collection, where the AI system needs data to learn from, such as pictures, text, or other types of information. After data collection, the data goes through data preparation, where it is cleaned, organized, and transformed into a suitable format for analysis (Yang and Talha, 2021). The cleaned and organized data is then used for model training, where the AI model learns from the data by recognizing patterns and relationships. During this stage, the AI model adjusts its parameters to make better predictions based on the data it has been trained on. Once the model is trained, it goes through model evaluation, where it is tested using separate data to assess its accuracy and reliability in making predictions. Finally, after successful model evaluation, the model is deployed in real-world applications to make predictions or perform tasks, as it is now considered accurate and reliable (Liu, 2021; Cao, 2023).

## 3. How AI can help in bone fracture detection

Developing and deploying an AI system for bone fracture detection involves several stages. The first stage is data collection, where a dataset of X-ray images of bone fractures is gathered from various sources, such as hospitals or medical databases. These images are labeled to indicate whether a fracture is present or not. Next, the collected X-ray images undergo data preparation, which involves preprocessing tasks such as resizing, normalizing pixel values, and augmenting the dataset with additional images to increase diversity.

Once the data is prepared, the AI model undergoes model training. The preprocessed X-ray images train the model, which learns to identify patterns and features indicative of bone fractures. The model adjusts its parameters through iterative training to minimize errors and improve accuracy by comparing its predictions with the labeled information in the dataset.

After the model is trained, it undergoes model evaluation. It is tested on a separate set of X-ray images not used in training. The model's accuracy, sensitivity, and specificity in detecting fractures are evaluated by comparing its predictions with the ground truth labels to assess its performance. Upon achieving satisfactory accuracy, the trained AI model can be deployed in a real-world clinical setting. It can be integrated into a software application or a medical imaging system to analyze X-ray images and provide automated fracture detection results. This can assist radiologists and physicians in accurately and efficiently diagnosing bone fractures, potentially improving patient care and outcomes (Meena and Roy, 2022). AI has the potential to enhance the diagnosis of actual scaphoid or hip fractures, identify significant fracture characteristics that can impact treatment and prognosis, and detect minor fractures that are frequently missed during a follow-up assessment after complex trauma (Guly, 2001).

## 4. Can AI replace orthopedic doctors in orthopedic radiography analysis?

The use of AI in bone fracture detection has shown promising results, particularly in detecting common fractures that humans and machines find relatively easy to detect. However, there are limitations to the current applications of AI in fracture detection.

One limitation is that the studies have mostly focused on common fractures, and more subtle fractures, such as no displaced femoral neck or scaphoid fractures, need further study to determine the accuracy of AI models in detecting them. Additionally, AI algorithms for diagnosing relatively obvious fractures might be useful in clinical scenarios where fractures might be overlooked or in primary care or urgent care where a radiologist is not immediately available.

Sometimes, diagnosing bone fractures may require integrating information from multiple imaging modalities, such as X-ray, CT scan, and MRI. Radiologists are trained to compare and correlate findings from various imaging modalities to provide a comprehensive diagnosis. AI algorithms that focus on a single modality may lack the ability to integrate information effectively from different imaging sources. Beyond fracture detection, orthopedic surgeons are vital in planning and executing appropriate treatment strategies. They consider factors like the type of fracture, its location, stability, and the patient's specific needs to determine the most suitable treatment option, whether conservative management or surgical intervention. The practice of medicine places a strong emphasis on patient-centered care, which involves considering the patient's values, preferences, and goals when making treatment decisions. Orthopedic surgeons and radiologists consider individual patient factors and engage in shared decision-making to ensure the best possible outcomes. AI algorithms cannot currently assess these factors comprehensively and make personalized treatment decisions.

However, clinicians might be reluctant to rely on AI suggestions due to the lack of a human interface, the complexity of statistical models, and the inscrutability of the “black box of AI.” Furthermore, there are liability concerns regarding who would be held responsible if an algorithm errors and causes harm. The European Union has addressed this concern by incorporating a dictum in the General Data Protection Regulations that AI algorithmic decisions about humans must be interpretable and explainable. Another limitation is the lack of reliable ground truth labels for training AI algorithms. Most studies used datasets with ground truth labels based on formal radiologist reports taken from the medical record, which have inherent errors and misinterpretations. Improved ground truth labels, such as operative findings or more sophisticated imaging, could lead to the development of more accurate AI algorithms. Moreover, an algorithm trained using such data might produce less precise forecasts concerning ethnic minorities, or any other group that is not well-represented in the dataset. This mirrors the bias found in human judgment, which arises from an individual's previous encounters, potentially resulting in incorrect clinical choices. Nonetheless, when a biased AI system is widely implemented and used by numerous clinicians at the same time, it can have even more harmful consequences for patient care and safety.

The current AI models developed for detecting bone fractures may not be able to identify other related abnormalities, such as tumors, infections, metabolic conditions, or inflammations that could explain a patient's symptoms or coexist with fractures (Link and Pedoia, 2022). Consequently, the AI system may generate a “fracture not found” result, leading to missed complications if another physician does not review the X-rays. Some of these complications can significantly impact patient outcomes and negatively affect their healthcare (Depypere et al., 2020). Therefore, a physician's supervision is always necessary when using AI in fracture detection. Moreover, AI systems usually do not estimate uncertainty or acknowledge the concept of “I do not know,” which can create a false sense of certainty and potentially lead to incorrect clinical decisions. It is crucial to be mindful of this limitation and carefully interpret the results produced by AI systems, considering the possibility of uncertainty and seeking expert medical judgment when making clinical decisions (Link and Pedoia, 2022). The current level of AI in detecting bone fractures is indeed impressive, particularly in high-traffic and low-resource locations (Wahl et al., 2018). However, it falls short of replacing orthopedic doctors completely due to its limitations. In summary, while AI technology can aid physicians in improving efficiency, it has not reached a point where it can entirely replace them.

## 5. Can AI replace radiologist in the future?

AI has shown promising results in detecting common fractures and could be helpful in clinical scenarios where fractures might be overlooked or in primary care or urgent care settings. However, some limitations need to be addressed, such as the accuracy of AI models in detecting more subtle fractures, liability concerns, and the need for improved ground truth labels for training AI algorithms. The management of bone fractures involves a range of treatment options, including surgery, medication, and physical therapy, and requires physicians with diagnostic abilities, clinical expertise, judgment, and communication skills to develop a comprehensive treatment plan tailored to each patient's needs Patients value the human connection and trust built with their doctors, which may not be replicable with AI systems. Moreover, AI systems used in healthcare are susceptible to cyber security threats and exploitation. The constant cat-and-mouse game in cyber security means that vulnerabilities can be discovered and exploited, potentially compromising the integrity and accuracy of AI-based fracture detection systems.

AI enhances radiology but can't fully replace radiologists due to interpretative skills, uncertainty, legal/ethical concerns, patient-clinician interaction, adaptability, bias, and trust. Instead, a collaborative future is envisioned where AI augments radiologists' expertise, assisting in radiography analysis. AI can preprocess images, segment structures, detect abnormalities, provide automated second opinions, prioritize cases, optimize workflows, and aid resource allocation. Despite its potential, AI's current capabilities cannot match the nuanced decision-making of radiologists. The risk of the system being compromised by a cyber security is also present. In short, full replacement of physicians is unlikely due to expertise, emotional intelligence, and ethical/legal considerations (Korot et al., 2020). Even in the foreseeable future, the likelihood of AI accomplishing such a feat remains exceedingly low.

Therefore, the ideal approach would be integrating AI as a tool to augment human decision-making and enhance healthcare delivery. At the same time, doctors continue to play a central role in clinical decision-making and patient care.

## 6. Conclusion: synergizing AI and medical expertise to enhance orthopedic radiography and patient care

The way AI is improving, we expect that AI can interpret more complex data to aid doctors in the future. Right now, it cannot help with multiple conditions like fractures, tumors, and inflammation. The use of AI algorithms in healthcare raises legal and ethical concerns. Liability issues can arise if an AI algorithm fails to detect a fracture or misinterprets an image, leading to a wrong diagnosis or delayed treatment. Establishing accountability and responsibility for such cases can be complex, especially in the absence of human expertise. As the AI is learning, we can expect better analysis and results with the data. Along with this, AI can also help in recommending treatment and surgical approaches depending on the data provided to it. It will be extremely beneficial for conditions in which a rare surgery is much better than its common alternative. When AI recommends the surgery, the surgeon can look into those options during their prognosis and surgical plan.

The emergence of advanced AI language models is revolutionizing the field of medicine, presenting new and exciting interventions that are transforming healthcare practices. According to Jeblick et al.'s case study, GPT-4 software like ChatGPT exhibits commendable proficiency in converting radiology reports into easily understandable language, achieving an average score of 4.27 on the five-point system. The study found only a minimal amount of missing information (0.08 places) and a slight presence of misinformation (0.07 places). Nevertheless, the study recognizes the need for continuous efforts to address the identified limitations and unlock the full potential of these models to optimize their applications in healthcare settings (Lyu et al., 2023).

Medical knowledge and practices are constantly evolving. New fracture types, classification systems, treatment techniques, and research findings emerge regularly. It is crucial for healthcare professionals to stay updated and adapt to these advancements. Orthopedics and radiologists undergo continuous training and professional development to ensure they provide the highest standard of care. Incorporating these advancements into AI algorithms and keeping them up to date would be challenging and time-consuming. Furthermore, orthopedicsurgeons and radiologists take into account individual patient factors and engage in shared decision-making to ensure the best possible outcomes. AI algorithms, lacking the ability to understand patient perspectives and preferences, cannot replicate this crucial aspect of patient care.

Radiologists who embrace and utilize AI technologies can enhance their diagnostic capabilities and improve patient care. The collaboration between AI and doctors holds promise for improving the overall healthcare experience for patients, but it should be viewed as a tool to support and enhance the expertise of healthcare professionals rather than a substitute for their skills and judgment. AI significantly enhanced the patient-wise sensitivity of radiologists for fracture detection by 20% and specificity by 0.6%. When working with AI assistance, radiologists demonstrated improved performance and efficiency compared to their unassisted counterparts (Canoni-Meynet et al., 2022). Therefore, if the question is “Will AI replace radiologists?”, the answer is No. Although if the question is rephrased to “Can radiologists who use AI replace radiologists who don't? The answer to that will be Yes (Langlotz, 2019).

## Author contributions

All authors listed have made a substantial, direct, and intellectual contribution to the work and approved it for publication.
